# Occurrence of brachylaimid trematodes, alive and shell-trapped, in sympatric species of terrestrial gastropods in France

**DOI:** 10.1051/parasite/2026052

**Published:** 2026-10-02

**Authors:** Claudia Gérard, Maxime Dahirel, Marie-Claire Martin, Armelle Ansart

**Affiliations:** 1 Université de Rennes, CNRS, ECOBIO (Ecosystèmes, Biodiversité, Evolution) – UMR 6553 35000 Rennes France; 2 Department of Biology, Ghent University B-9000 Ghent Belgium

**Keywords:** Sympatric land snails, Metazoan parasites, Larval trematodes, *Brachylaima*, *Pseudoleucochloridium*, New records

## Abstract

Following the detection of *Brachylaima* spp., which are potentially pathogenic for domestic animals and humans, in *Cornu aspersum* and *Cepaea nemoralis* at a rural site in France, we investigated trematode distribution among sympatric gastropod species to assess their role in the parasite life cycle. Eight species were investigated (*Cepaea nemoralis*, *Cernuella neglecta, Clausilia bidentata*, *Cochlicella acuta, Cornu aspersum*, *Lauria cylindracea, Pomatias elegans*, and *Theba pisana*). Four species were parasitised by trematodes: *C. aspersum* (prevalence 56%), *C. nemoralis* (10%), and *T. pisana* (2%) as second intermediate hosts of *Brachylaima* spp., and *C. neglecta* (6%) as new first intermediate host of *Pseudoleucochloridium* sp. Moreover, we observed for the first time shell-encapsulation of trematodes in a non-helicid species (*C. neglecta*) and nematodes in a non-stylommatophoran species (*P. elegans*), suggesting more general anti-parasite defence than previously thought among land snails. Our current and previous results, including molecular data (28S rDNA, COI mtDNA), suggest the occurrence of four *Brachylaima* species at the site: *Brachylaima mesostoma* and three unidentified species. *Cornu aspersum* is the only identified first intermediate host for *B. mesostoma* and one of the unidentified trematode species also found at a metacercarial stage in *C. aspersum* and *C. nemoralis*. Another unidentified species recorded in *C. aspersum* and *C. nemoralis*, characterised by small orange metacercariae, may be a new species. The sole metacercaria found in *T. pisana* may also belong to an undescribed species.

## Abstract (fr)

Suite à la détection de *Brachylaima* spp., qui sont potentiellement pathogènes pour les animaux domestiques et les humains, chez *Cornu aspersum* et *Cepaea nemoralis* sur un site rural français, nous avons étudié la répartition des trématodes parmi des espèces de gastéropodes sympatriques afin d'évaluer leur rôle dans le cycle biologique des parasites. Huit espèces ont été étudiées : *Cepaea nemoralis*, *Cernuella neglecta, Clausilia bidentata*, *Cochlicella acuta, Cornu aspersum*, *Lauria cylindracea, Pomatias elegans* et *Theba pisana*. Quatre espèces étaient parasitées par des trématodes : *C. aspersum* (prévalence 56 %), *C. nemoralis* (10 %) et *T. pisana* (2 %) comme seconds hôtes intermédiaires de *Brachylaima* spp., et *C. neglecta* (6 %) comme nouvel hôte intermédiaire primaire de *Pseudoleucochloridium* sp. Par ailleurs, nous avons observé pour la première fois l'encapsulation de trématodes dans la coquille d'un non-Hélicidé (*C. neglecta*) et de nématodes dans celle d'un non-Stylommatophore (*P. elegans*), ce qui suggère l'existence d'un mécanisme de défense antiparasitaire plus général chez les escargots terrestres qu'on ne le pensait auparavant. Nos résultats actuels et antérieurs, incluant des données moléculaires (ADNr 28S, ADNmt COI), indiquent la présence de quatre espèces de *Brachylaima* sur le site : *Brachylaima mesostoma* et trois espèces non identifiées. *Cornu aspersum* est le seul hôte intermédiaire primaire identifié pour *B. mesostoma* ainsi que pour l'une des espèces de trématodes non identifiées, également retrouvée au stade métacercaire chez *C. aspersum* et *C. nemoralis*. Une autre espèce non identifiée, observée chez *C. aspersum* et *C. nemoralis* et caractérisée par de petites métacercaires orange, pourrait constituer une nouvelle espèce. L'unique métacercaire trouvée chez *T. pisana* pourrait également appartenir à une espèce non décrite.

## Introduction

Brachylaimiasis, an intestinal parasitosis induced by haematophagous digenean trematodes of the genus *Brachylaima* Dujardin, 1843, is a potentially fatal infection in domestic vertebrates, with, for example, reports of mortality in pigs and dogs with high parasite burdens [3, 19, 27, 38]. Until now, human brachylaimiasis, causing symptoms such as diarrhoea, abdominal pain, and anorexia, has been described exclusively in Australia and was found to be caused by *Brachylaima cribbi* Butcher and Grove, 2001 [7–9, 13]. Infection occurs after the consumption of raw or undercooked land snails parasitised by metacercarial larvae, but also after the ingestion of live metacercariae deposited on vegetables and fruits via the snails’ slime and excreta and/or crushed infected snails [7, 8, 11, 13]. Diagnosis of brachylaimiasis is based on the detection of eggs in human faeces, but the search for *Brachylaima* eggs is not included in standard medical protocols of helminth detection [13, 42, 58], potentially resulting in misdiagnosis and underestimation of infected people worldwide.

*Brachylaima cribbi* is considered native to Europe because its most commonly recorded hosts are European species introduced to Australia: land snails including *Cochlicella* sp., *Theba pisana*, and *Cornu aspersum* (first and second intermediate hosts), European blackbird *Turdus merula* and starling *Sturnus vulgaris* (definitive hosts) [9, 10, 21]. Its host range is, however, more diverse and also includes other birds, mammals, and even lizards, both native and introduced [11, 12, 21]. Beyond Australia and *B. cribbi* specifically, commercial trade of edible gastropods has been shown to be a potential vector for the intercontinental spread of other *Brachylaima* species (between Spain and both North and Southern Africa [31]). In Europe, at least 12 species of *Brachylaima* (including potentially cryptic species) have been recorded, with likely more to be discovered, infecting a wide range of terrestrial gastropods and vertebrates (Table 1). Despite the probable European origin of *B. cribbi*, its occurrence has not yet been demonstrated in Europe, possibly due to a lack of field investigations.

**Table 1:** Intermediate (first and second Hi) and definitive (Hd) hosts of *Brachylaima* spp. recorded in Europe according to bibliographical data.

*Brachylaima* species	1st Hi	2nd Hi	Hd	References
*Brachylaima arcuatus* (Dujardin, 1845)	Unknown	*Ambigolimax valentianus*, *Deroceras invadens*, *D. reticulatum*, *Monacha cantiana*, *Tandonia sowerbyi*	Birds (Corvidae, Sylviidae, Turdidae)	[2, 25, 41]
*Brachylaima aspersae* Segade *et al.*, 2011	*Cornu aspersum*	*Cornu aspersum*	Rodents (e.g. *Mus* sp.)	[35]
*Brachylaima erinaceus* Blanchard, 1847	*Agriolimax reticulatus*, *Helicella ericetorum*, *H. variabilis*	*Agriolimax reticulatus*, *Helicella ericetorum*, *H. variabilis*	Mammals (badger, cat, dog, hedgehog)	[25, 27]
*Brachylaima fulvus* Dujardin, 1843	Unknown	*Goniodiscus rotundatus*, *Zonitoides excavatus*, *Z. nitidus*	*Sorex* sp., *Neomys fodiens*	[25, 43, 46, 60]
*Brachylaima fuscata* (Rudolphi, 1819)	*Agriolimax agrestis*, *Cochlicella ventrosa*, *Cornu aspersum*, *Helicella profuga*, *Hygromia cinctella*, *Theba pisana*	*Agriolimax agrestis*, *Ambigolimax valentianus*, *Cepaea hortensis*, *Cochlicella ventrosa*, *Cornu aspersum*, *Deroceras invadens*, *Helicella profuga*, *Hygromia cinctella*, *Oxychilus cellarius*, *Theba pisana*, *Trochulus striolatus*	Birds (Accipitridae, Columbidae, Corvidae, Laridae, Passeridae, Phasianidae, Strigidae, Sylviidae, Turdidae)	[2, 24, 36, 41, 72]
*Brachylaima llobregatensis* González-Moreno and Gracenea, 2006	*Cornu aspersum*	*Cornu aspersum*, *Otala punctata*, *Theba pisana*	Rodents (*Crocidura russula, Mus* sp*.*)	[35]
*Brachylaima mascomai* Gracenea and González-Moreno, 2002	*Pseudotachea splendida*	*Cornu aspersum*, *Otala punctata*, *Pseudotachea splendida*, *Theba pisana*	Rodents (*Apodemus sylvaticus*, *Mus* sp., *Rattus* sp*.*)	[36]
*Brachylaima mesostoma* (Rudolphi, 1803)	*Cornu aspersum*, *Cepaea hortensis*, *C. nemoralis*, *Xeropicta krynickii*	*Brephulopsis bidens*, *B. cylindrica*, *Cepaea hortensis*, *C. nemoralis*, *Cornu aspersum*, *Eobania vermiculata*, *Helicopsis filimargo*, *H. retowskii*, *Thoanteus gibber*, *Xeropicta krynickii*	Birds (Corvidae, Fringillidae, Phasianidae including *Gallus domesticus*, Sylviidae, Turdidae)	[2, 32, 41, 66, 73]
*Brachylaima nicolli* (Wintenberg, 1925)	Unknown	*Agriolimax agrestis*, *Helicella scitula*, *Oxyphilus cellarius*	Birds (Passeridae)	[18]
*Brachylaima recurva* (Dujardin, 1845)	*Cepaea hortensis, Cornu aspersum*	*Cepaea hortensis*, *C. nemoralis*, *Cornu aspersum*, *Fruticicola fruticum*, *Helix pomatia*, *Pomatias elegans*	Mammals (*Apodemus sylvaticus*, *Eliomys quercinus*, *Vulpes vulpes*)	[24, 25, 53, 63, 68]
*Brachylaima ruminae* Mas-Coma and Montoliu, 1986	*Rumina decollata*	*Ferrussacia follicula*, *Rumina decollata*	Rodents (*Eliomys* sp., *Mus* sp., *Rattus* sp.)	[50]
*Brachylaima suis* Balozet, 1936	Helicidae (*Cornu* sp., *Helix* sp., *Theba* sp.)	Helicidae (*Cornu* sp., *Helix* sp., *Theba* sp.)	Mammals (domestic and wild pigs, rodents)	[3, 19, 27]

Molecular sequences of *Brachylaima* spp. are rarely available and taxonomic confusion reigns among the family of Brachylaimidae [70].

In recent years, there has been growing global interest in protein sources other than vertebrate meat, and land snails are emerging as an attractive alternative due to their high content of high-quality protein, low fat and cholesterol content, and their supply of essential fatty acids, amino acids, and minerals ([39, 40, 67] for reviews). Commercial snail trade is also on the rise due to the increasing use of snail slime in various areas such as medicine, cosmetics, and biotechnologies ([40, 47, 62] for reviews). France is one of the leading countries worldwide for consumption of land gastropods (*C. aspersum*, *Helix pomatia*, and *Helix lucorum*), with about 17,000 tons ingested per year in alive snails-equivalent, either gathered in the wild or sourced from heliciculture farms [26]. Most snails are imported from 28 foreign countries (mainly Romania, Belgium, and Türkiye) as farms in France produce only about 1,000 tons of snails per year [26].

Despite the diversity of *Brachylaima* species in Europe and the interactions between their potential snail hosts and humans or domestic animals, we know little about their host spectra and the potential impacts of metacercariae ingestion on vertebrate definitive hosts, or the health consequences in humans. In this context, better characterisation of the diversity of *Brachylaima* species and the gastropod species that may serve as a reservoir is needed. We previously found *Brachylaima* spp. infections in studies focused on the common helicid snails *C. aspersum* and *Cepaea nemoralis*, in and around a rural village in France [22, 32]. Here, we revisited the same area to investigate the distribution of trematode larvae in the broader shelled gastropod community in order to assess how sympatric land snail species are involved in the life cycle and transmission of *Brachylaima*.

## Materials and methods

### Ethics

This study was carried out in compliance with all relevant international and national laws; none of the species considered is formally protected, no ethics committee recommendation nor administrative authorization was needed to sample or study any of the snail or parasite species mentioned here.

### Study site, sampling, and measurements

We sampled wild sympatric populations of land snails on September 10–11, 2022, in a private garden in a rural area (northwestern France, Arçais, approx. 46°17′50″ N, 0°41′30″W) where we previously (April 2018) recorded the occurrence of *Brachylaima* spp. sporocysts and metacercariae in *C. aspersum* [32]. *Brachylaima* metacercariae were also found in *C. nemoralis* from Arçais in May 2019 [22].

We sampled by hand a total of 404 adults belonging to eight gastropod species (*C. nemoralis*, *Clausilia bidentata*, *Cernuella neglecta*, *Cochlicella acuta*, *C. aspersum*, *Lauria cylindracea*, *T. pisana*, and *Pomatias elegans*; Table 2). The height and width of the snail shell were measured with a caliper (precision: 0.1 mm). Depending on the species, we identified adult gastropods based on the size of their shell and/or the presence of a differentiated peristome (reflected “lip” around the shell opening or thickened inner rim indicating the cessation of shell growth [59]).

**Table 2:** Taxonomy, number (*N*) and shell size (height and width ± CI, in mm) of adult terrestrial gastropods belonging to eight species sampled in a private garden (Arçais, France) in September 2022 with information on their main diet, microhabitat, vertebrate predators, and lifespan.

	*N*	Height ± CI	Width ± CI	Diet	Microhabitat*	Vertebrate predators**	Lifespan
				[29]	[29, 71]	[1, 45]	[29]
**SubClass-Order: HETEROBRANCHIA – STYLOMMATOPHORA**							
**SuperFam.: Helicoidea**							
**Fam.: Helicidae**							
**Sub.Fam.: Helicinae**							
*Cepaea nemoralis*	50	19.02 ± 0.39	24.07 ± 0.34	Living and dead higher plants	Among/under vegetation	Birds (Muscicapidae, Phasianidae, Turdidae); mammals (Erinaceidae, Soricidae, Suidae); reptiles (Anguidae)	2–5 yrs
*Cornu aspersum*	50	29.39 ± 0.47	29.58 ± 0.58	Living and dead higher plants, saprophagous	Among/under vegetation and surface debris	Birds (Turdidae, Anatidae); mammals (Erinaceidae, Muridae, Soricidae, Talpidae); reptiles (Anguidae)	2 – > 5 yrs
*Theba pisana*	50	14.26 ± 0.36	18.83 ± 0.30	Living and dead higher plants	Among/under vegetation, climbs on vertical surfaces in hot weather	Birds (Turdidae, Anatidae, Corvidae, Falconidae); mammals (Erinaceidae, Muridae, Soricidae)	1–2 yrs
**Fam: Geomitridae**							
**SubFam.: Geomitrinae**							
*Cochlicella acuta*	51	15.62 ± 0.42	6.37 ± 2.12	Dead higher plants	Herb layer, among/under vegetation, climbs on vertical surfaces in hot weather	Birds (Corvidae, Falconidae)	1–2 yrs
**SubFam.: Helicellinae**							
*Cernuella neglecta*	51	9.52 ± 0.22	14.45 ± 0.26	Dead higher plants	Shrubs/bushes/saplings and among/under vegetation on ground, climbs on vertical surfaces in hot weather	Birds (Turdidae); mammals (Muridae)	1–2 yrs
**SuperFam.: Clausilioidea**							
**Fam.: Clausiliidae**							
**Sub.Fam.: Clausiliinae**							
*Clausilia bidentata*	50	10.50 ± 0.28	2.62 ± 0.12	Living algae, lichens, dead higher plants	In/under timber, among/under surface debris	Birds (Turdidae)	2–5 yrs
**SuperFam.: Pupilloidea**							
**Fam: Lauriidae**							
*Lauria cylindracea*	50	3.72 ± 0.10	–	Dead higher plants, forest litter	In/under timber, among/under surface debris, under stones	–	1–2 yrs
**SubClass-Order: CAENOGASTROPODA – LITTORINOMORPHA**							
**SuperFam.: Littorinoidea**							
**Fam.: Pomatiidae**							
*Pomatias elegans*	52	13.69 ± 0.21	9.52 ± 0.19	Dead higher plants, forest litter	Among/under surface debris, dig into the substrate to hibernate and estivate	Mammals (Erinaceidae)	2 – > 5 yrs

*All species are epigean, present on plants and on ground surface.

**Only predators described in Western Europe are considered.

The collected snails were immediately frozen and stored at −20 °C until they were examined. After thawing, we dissected each snail using a binocular stereoscopic microscope with backlighting. After removing the body from the shell, we searched for metazoan parasites in the shell (encysted specimens, see Rae [61] and Gérard *et al.* [33] for more information) and in various locations (i.e., space between shell and soft body, lung, heart, kidney, body cavity, genital and digestive systems), as in previous studies [22, 32, 33]. We found larval trematodes, nematodes, and parasitic mites (Acari). When present, trematodes (sporocysts, cercariae, and/or metacercariae) were observed under light glass coverslip pressure using bright-field and phase-contrast microscopy, and were counted. We morphologically identified metacercariae of the genus *Brachylaima* according to Gracenea and González-Moreno [36] and Segade *et al.* [65]. We performed measurements of metacercariae preserved in 95% ethanol, following Mas-Coma *et al.* [49], and we also preserved sporocysts and metacercariae in 95% ethanol at −20 °C for DNA sequence analysis (see below).

We described parasitism by prevalence (P%) (number of hosts infected with a particular parasite species/number of examined hosts) and mean intensity (I) (total number of parasites of a particular species found in a sample/number of hosts infected with that parasite) [6].

We report mean values followed by the 95% confidence interval (CI). For prevalence, we calculated CI using the score method [57].

### DNA sequencing and identification of trematodes

Three sporocyst samples from *C. neglecta* and 13 individual metacercariae (seven from *C. nemoralis*, five from *C. aspersum*, one from *T. pisana*) of trematodes, each of them originating from a different snail [except for two *C. nemoralis* in which two morphologically distinct forms of metacercaria (orange or not) were found], were analysed by molecular identification tools. DNA of each individual parasite (approximately 1 mm^3^ piece of sporocyst or whole body of a metacercaria) was extracted following Nakao *et al.* [55]. Samples were lysed in 25 μL of 0.02 N NaOH at 99 °C for 30 min. Unfortunately, insufficient DNA was obtained for the sole metacercaria found in *T. pisana*, requiring us to remove this sample from further analyses. We amplified fragments for the nuclear 28S ribosomal DNA (rDNA) and mitochondrial COI DNA (mtDNA) genes, respectively as presented in Gérard *et al.* [32]. The 28S fragment was amplified using the forward primer dig12 (5′–AAGCATATCACTAAGCGG–3′) and the reverse primer 1500R (5′–GCTATCCTGAGGGAAACTTCG–3′) [69]. The COI region was amplified using the forward primer JB3 (5′–TTTTTTGGGCATCCTGAGGTTTAT–3′) and the reverse primer CO1-R trema (5′–CAACAAATCAT GATGCAAAAGG–3′) [52]. Amplification of template DNA was carried out with MyTaq Mix (2X) (Bioline, France), including 5 μM of each primer and between 2 and 5 μL of DNA depending on its concentration. The reaction mixture was made up to a total volume of 25 μL with ultrapure water. The PCR conditions were 98 °C for 10 s, 50 °C for 20 s, and 68 °C for 90 s (40 cycles). We checked amplification products using a 2% agarose gel stained with ethidium bromide. We obtained double-strand sequences with an automated sequencer (Plateforme de séquençage et génotypage OUEST-génopole). Unless we performed several dilutions of DNA extracts to discard contaminants appearing to be abundant in our samples, we obtained overall poor quality sequences, especially for rDNA 28S fragments. We had to shorten them or to discard some from further analysis. Editing and correction of sequences were performed using BioEdit Sequence Alignment Editor software, version 7.7.1. Coding sequences (mtDNA COI) were determined with the on-line tool ORF Finder (NCBI). In the end, we obtained 12 28S sequences and 5 COI sequences useable for parasite identification. The BLAST Analysis Tool from the NCBI was used to examine the similarity between these sequences and the publicly available references.

## Results

### 1) Trematode infections

Among the 404 adult snails collected, 37 were parasitised by larval trematodes (prevalence of 9.2% [CI 6.7–12.4%]), all belonging to the family of Brachylaimidae. These parasites were recorded in four of the eight terrestrial gastropod species, in decreasing order of prevalence: *C. aspersum*, *C. nemoralis*, *C. neglecta*, and *T. pisana* (Table 3).

**Table 3:** Prevalence (P% [CI]) and mean intensity (I ± CI) of larval Brachylaimidae (Spo = sporocysts, Meta = metacercariae) in terrestrial gastropods (all adults) from Arçais in September 2022. Two *C. nemoralis* individuals had mixed infections with both *Bra1* and *Bra2* metacercariae in their kidney.

	*Pseudoleucochloridium* sp.		*Brachylaima* sp.1 (*Bra1*)**		*Brachylaima* sp.2 (*Bra2*)	
Snail species	P% Spo	I Spo *(range)*	P% Meta	Snail species	P% Spo	I Spo *(range)*
*Cepaea nemoralis*	0	0	8.0 [3.2–18.8]	5.00 ± 1.60 *(4*–*6)*	6.0 [2.1–16.2]	7.00 ± 11.02 *(1*–*13)*
*Cornu aspersum*	0	0	54.0 [40.4–67.0]	23.41 ± 11.21 *(1*–*117)*	2.0 [0.4–10.5]	8
*Theba pisana*	0	0	2.0 [0.4–10.5]	1	0	0
*Cochlicella acuta*	0	0	0	0	0	0
*Cernuella neglecta*	5.9 [2.0–15.9]*	68.33 ± 100.76 *(5*–*100)*	0	0	0	0
*Clausilia bidentata*	0	0	0	0	0	0
*Lauria cylindracea*	0	0	0	0	0	0
*Pomatias elegans*	0	0	0	0	0	0

*One unparasitised adult of *C. neglecta* had two brachylaimid metacercariae encysted into the shell (P% = 2.0 [0.4–10.3]).

**Brachylaima sp.1 metacercariae of *C. aspersum* and *C. nemoralis* corresponded to the unidentified *Brachylaima* sp*.* in *C. aspersum* [32].

### 2) Morphological and genetic identification of trematode larvae

#### a – Metacercariae of *Brachylaima* spp. in *C. aspersum*, *C. nemoralis*, and *T. pisana*

The total prevalence of *Brachylaima* spp. was 56.0% (CI 42.3–68.8%) for *C. aspersum*, 10.0% (4.3–21.4%) for *C. nemoralis*, and 2.0% (0.4–10.5%) for *T. pisana* (a single infected individual), with mean intensities of 22.86 ± 10.84 (range: 1–117 metacercariae), 8.20 ± 5.97 (range: 4–17), and 1 metacercaria, respectively.

Morphological characteristics of metacercariae allowed discrimination of two morphospecies of *Brachylaima*, named *Brachylaima* sp.1 (*Bra1*) and *Brachylaima* sp.2 (*Bra2*) below.

Bra1 was the most frequently recorded in our study and found in the kidney of the three helicid species, *i.e.*, 28 *C. aspersum*, four *C. nemoralis*, and one *T. pisana* (Table 3). Metacercariae were morphologically similar across host species, with caeca containing numerous urate concretions from ingested renal epithelium, but the smaller size of the single metacercaria found in *T. pisana* (total length = 1,330 μm; Fig. 1A) compared to those of *C. aspersum* and *C. nemoralis* (2,127 ± 125 μm, range of 1,739–2,430 μm) may be indicative of another *Brachylaima* species (named *Bra1-Tp*).

**Figure 1:**
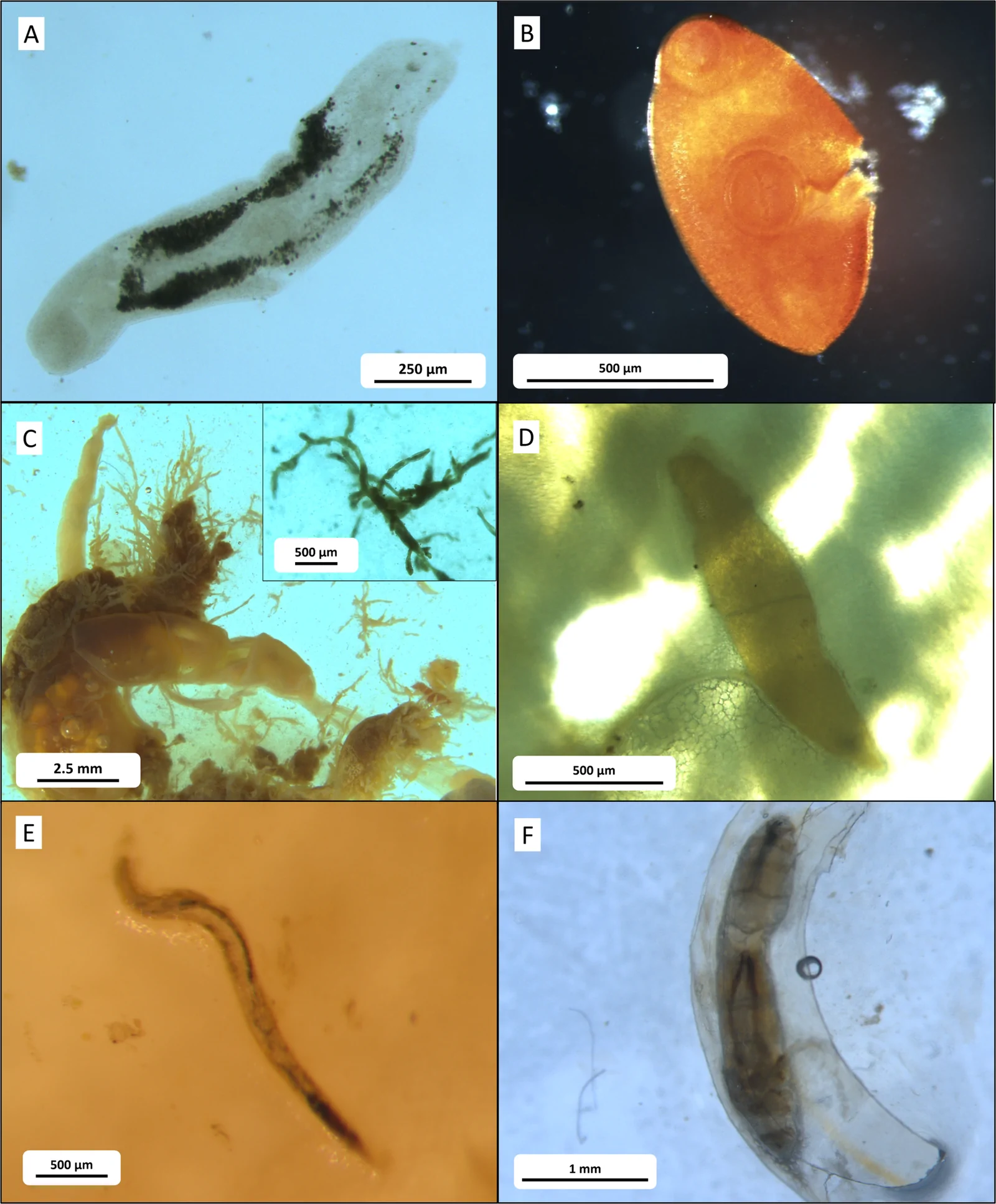
A, B: Metacercariae of *Brachylaima* infecting the helicid kidney, with A: the only metacercaria of *Bra1-Tp* found in one *Theba pisana* (total length = 1,330 μm, total width = 280 μm; note that caeca contain numerous urate concretions from ingested renal epithelium, also observed for most *Bra1* metacercariae in *C. aspersum* and *C. nemoralis*), and B: Orange metacercaria *Bra2* found in *Cepaea nemoralis* (total length = 975 μm, total width = 543 μm); C: Branched cercariogenous sporocyst of *Pseudoleucochloridium* sp. in the digestive gland of *Cernuella virgata* (general view of the parasitised digestive gland and inserted sporocyst detail); D: Brachylaimidae metacercaria encapsulated in the shell of one uninfected *Cernuella virgata*; E: Nematode encapsulated in the shell of *Pomatias elegans*; F: Encysted dipteran larva in the shell of *Theba pisana*.

Following Moszczynska *et al.* [54] and Blasco-Costa *et al.* [5], we considered genetic species identification mainly based on COI comparisons for which query-cover (proportion of alignment between both sequences) was over 90%, E-value (expected number of matches of similar quality that would occur by chance) close to 0 and an Identity (percentage of nucleotides that are identical in the aligned region) of at least 97%. On these criteria, we were able to match the successfully sequenced *Bra1* metacercariae (in four *C. nemoralis* and two *C. aspersum* individuals) to the same unnamed *Brachylaima* species we previously characterised from two samples collected in April 2018 (one sporocyst from the same site, and one metacercaria from another [32]; Table 4). Unfortunately, DNA isolation of the single metacercaria found in *T. pisana* (*Bra1-Tp*), possibly belonging to a different species, was unsuccessful.

**Table 4:** Identification of parasite species using BLAST (NCBI), based on COI mtDNA sequences obtained from metacercariae corresponding to the *Bra1* morphotype and collected from the land snails *Cepaea nemoralis* (four individuals) and *Cornu aspersum* (two individuals) in September 2022 in Arçais.

Analysed sequence			BLAST results*			GenBank sample	
Snail species	Parasite larval stage	Sample ID	query cover (%)	E-value	Identity (%)	Accession number	Parasite species
*Cepaea nemoralis*							
	metacercaria	Cn23	100%	0.0	99.77	MN848408.1**	*Brachylaima* sp.
			100%	0.0	97.42	MN849923.1**	*Brachylaima* sp.
							
	metacercaria	Cn30	100%	0.0	98.38	MN848408.1	*Brachylaima* sp.
							
	metacercaria	Cn42	99%	0.0	99.11	MN848408.1	*Brachylaima* sp.
							
	metacercaria	Cn45	100%	0.0	99.78	MN848408.1	*Brachylaima* sp.
			100%	0.0	97.60	MN849923.1	*Brachylaima* sp.
*Cornu aspersum*							
	metacercaria	Ca5	98%	0.0	99.62	MN848408.1	*Brachylaima* sp.
	metacercaria	Ca47	100%	5.0^−103^	100.00	MN849923.1	*Brachylaima* sp.
			100%	5.0^−103^	100.00	MN848408.1	*Brachylaima* sp.

*Only matches for which query-cover >90%, E-value <10^−6^ and Identity >97% are considered.

**Sequences from our previous study: please see [32].

***Bra2*** was rare and only present in three adults of *C. nemoralis* and one of *C. aspersum* (Table 3). Among them, mixed infections with *Bra1* metacercariae were detected in two *C. nemoralis* individuals. *Bra2* metacercariae were orange and smaller (total length = 815 ± 207 μm, range of 520–1,050 μm; Fig. 1B) than *Bra1*. COI comparisons with our last sequences of *Brachylaima* sp. suggest that it belongs to the genus *Brachylaima*, but probably to a different species (MN848408.1: query cover 70%, E-value 4.0^−108^, Identity 94.27%; MN849923.1: query cover 66%, E-value 2.0^−106^, Identity 95.55%).

#### b – Sporocysts of *Pseudoleucochloridium* sp. in *C. neglecta*

Three snails among the 51 adults of *C. neglecta* were found to be infected with branched cercariogenous sporocysts (Fig. 1C; Table 5). One infected snail had only a few sporocysts in its digestive and genital glands, while the digestive gland of the other two snails was completely invaded by sporocysts. Sporocysts found in the three *C. neglecta* individuals could not be identified to the species level. However, both genetic markers (COI and 28S) matched with the same samples originating from *Pseudoleucochloridium ainohelicis* Nakao, Sasaki, Waki & Asakawa, 2019, a Brachylaimidae found in Japan and using the land snail *Ainohelix editha* as first intermediate host [56] (Table 5). Although the percent of Identity was quite low for the COI sequences (from 83.55 to 85.11%), comparisons with 28S sequences yielded high and similar results for the three samples (query cover 100%, E-value 0.0, Identity between 98.22% and 98.66%), suggesting that they belong to the genus *Pseudoleucochloridium* Pojmańska, 1959.

**Table 5:** Identification of parasite genus using BLAST (NCBI), based on COI mtDNA and 28S rDNA sequences obtained from sporocysts collected from the land snail *Cernuella neglecta* (three individuals) in September 2022 in Arçais.

Analysed sequence			BLAST results*			GenBank sample	
Parasite larval stage	Sample ID	Genetic sequence	Query cover (%)	E-value	Identity (%)	Accession number	Parasite species
Sporocyst	Ce10	COI	82%	6.0^−157^	83.55	LC455745.1**	*Pseudoleucochloridium ainohelicis*
		28S	100%	0.0	98.66	LC455740.1**	*Pseudoleucochloridium ainohelicis*
							
Sporocyst	Ce21	COI	96%	0.0	84.78	LC455745.1	*Pseudoleucochloridium ainohelicis*
		28S	100%	0.0	98.66	LC455740.1	*Pseudoleucochloridium ainohelicis*
							
Sporocyst	Ce22	COI	75%	2.0^−152^	85.11	LC455745.1	*Pseudoleucochloridium ainohelicis*
		28S	100%	0.0	98.22	LC455740.1	*Pseudoleucochloridium ainohelicis*

*First occurrence from BLAST.

**Sequences from [56].

Interestingly, one uninfected adult of *C. neglecta* displayed two encapsulated Brachylaimidae metacercariae in its shell, with lengths of 1,388 μm and 1,420 μm, respectively (Fig. 1D).

### 3) Occurrence of other metazoan parasites: nematodes and mites (including dead shell-encapsulated)

We recorded nematodes in the lung of five helicid individuals: *C. aspersum* (three), *C. nemoralis* (one), and *T. pisana* (one) (Table 6). The three *C. aspersum* individuals infected by nematodes also harboured *Brachylaima* metacercariae in their kidney. We also found one mite (*Riccardoella* sp.) in the lung of one *C. nemoralis* specimen.

**Table 6:** Prevalence (P% [CI]) and mean intensity (I ± CI) of nematodes (“alive” in the lung and “dead” encapsulated in the shell) recorded in land snail species from a private garden (Arçais, France), based on this study, Dahirel *et al.* [22] and Gérard *et al.* [32].

Snail species	Nematodes in the lung		Shell-encapsulated Nematodes		References
	P% [CI]	I ± CI (range)	P% [CI]	I ± CI (range)	
*Cepaea nemoralis*					
May 2019	15.6 [CI 11.0–21.6]	1.86 ± 0.80 (1–9)	76.7 [CI 70.0–82.3]	11.67 ± 2.55 (1–88)	[22]
Sep 2022	2.0 [CI 0.4–10.5]	1	62.0 [CI 48.2–74.1]	2.87 ± 0.57 (1–14)	This study
*Cornu aspersum*					
Apr 2018	34.8 [CI 29.4–40.6]	3.16 ± 0.71 (1–18)	33.0 [CI 27.7–38.7]	2.66 ± 0.52 (1–16)	[32]
Sep 2022	6.0 [CI 2.1–16.2]	2.33 ± 2.12 (1–3)	62.0 [CI 48.2–74.1]	7.39 ± 2.89 (1–41)	This study
*Theba pisana*					
Sep 2022	2.0 [CI 0.4–10.5]	1	2.0 [CI 0.4–10.5]	1	This study
Pomatias elegans*					
Sep 2022	*–*	*–*	7.7 [CI 3.0–18.2]	3.75 ± 2.86 (1–6)	This study

*Red-brown nematodes encapsulated in *P. elegans*.

Moreover, nematodes were frequently found encapsulated (dead) in the shell of the three helicid species, mainly in *C. nemoralis* (31 individuals) and *C. aspersum* (31), and were discovered for the first time in the shell of the prosobranch *P. elegans* (four snails) (Table 6, Fig. 1E). We observed two *T. pisana* specimens with a dipteran larva encysted in the shell (Fig. 1F).

## Discussion

Apart from *C. aspersum* and *C. nemoralis*, sympatric terrestrial gastropod species in Arçais were rarely (*C. neglecta* and *T. pisana*) or not (*C. bidentata*, *C. acuta*, *L. cylindracea*, and *P. elegans*) infected by trematodes, nor by other live metazoan parasites such as nematodes and mites (Tables 3 and 6). Regarding trematodes, four out of eight snail species were found to be parasitised by two genera of Brachylaimidae: *Pseudoleucochloridium* (sporocysts, first intermediate host) in *C. neglecta* and *Brachylaima* (metacercariae, second intermediate hosts) in *C. aspersum*, *C. nemoralis*, and *T. pisana*, respectively. Moreover, our results suggest that shell-encapsulation may be a generalist defence response of land snails against natural enemies.

### *Pseudoleucochloridium* sp. / *C. neglecta*

In Europe, recorded parasites of *C. neglecta* comprise nematodes such as *Neostrongylus linearis* (Marotel, 1913) Gebauer, 1932 [64], *Phasmarhabditis elegans* (Schneider, 1859) [4], and trematodes such as *Dicrocoelium dendriticum* (Rudolphi, 1819) [30, 48]. There are also records in Australia, where *C. neglecta* is an introduced invasive species, with documented *B. cribbi* infections (both sporocysts and metacercariae) [10–12]. The present study is to our knowledge the first record of *Pseudoleucochloridium* sporocysts parasitizing *C. neglecta*. As far as is known, only five species have been described from the genus *Pseudoleucochloridium*, all recorded in the adult stage from shrews in Eurasia [14, 51, 56]. Among them, only *P. ainohelicis*, a Japanese species using the land snail *Ainohelix editha* as first intermediate host, has been sequenced [56]. Our results suggest that the sporocysts we found in *C. neglecta* belong to a closely related but different species from *P. ainohelicis*. This is all the more probable given the Japanese distribution of *P. ainohelicis* and the strict specificity of *Pseudoleucochloridium* spp. for the first intermediate host [51, 56]. In Western Europe (French Pyrenees), *Pseudoleucochloridium pericardicum* Mas-Coma and Montoliu, 1996 has been recorded in *Cepaea hortensis* as the specific first intermediate host. The second intermediate host was mainly another land snail, *Euomphalia strigella* (Hygromiidae), but *C. hortensis* and *C. aspersum* may to a lesser extent also play this role; in all cases, metacercariae are specifically located in the pericardium [37, 44, 51]. Three other species of *Pseudoleucochloridium* have been recorded in Eastern Europe, *i.e.*, *Pseudoleucochloridium rotundus* Bychowskaja-Pavlovskaja and Kulakova, 1970 in Ukraine, *Pseudoleucochloridium soricis* (synonym of *P. pericardicum* according to Mas-Coma & Montoliu [51]) in Poland, and *Pseudoleucochloridium skrjabini* Shaldibin, 1953 in Mordovia, Russian Federation, but the first intermediate host is unknown as well as most second intermediate hosts [14, 56]. In our study, none of the seven other snail species studied harboured *Pseudoleucochloridium* sporocysts or metacercariae, not even *C. nemoralis*. Given the low prevalences in *C. neglecta* and our relatively limited sample sizes per snail species, we cannot, however, exclude the possibility that these non-detections (especially in *C. nemoralis*) are a statistical artifact.

Interestingly, we also demonstrated for the first time the encapsulation of two Brachylaimidae metacercariae in the shell of one unparasitised *C. neglecta* adult. Based on their size (roughly 1,400 μm), these trapped metacercariae possibly belong to one of the Brachylaimidae species found in Arçais such as *Brachylaima* sp. recorded in *T. pisana* (*Bra1-Tp* metacercaria of 1,330 μm). Further research is needed to identify shell-encapsulated brachylaimid metacercariae and the species of *Pseudoleucochloridium* found in *C. neglecta*.

### *Brachylaima* spp. / *C. aspersum*, *C. nemoralis*, *T. pisana*

Only three out of the eight sympatric land snail species studied in Arçais were found to be infected by metacercariae of the genus *Brachylaima*, and may have a role in the transmission of potentially harmful *Brachylaima* to vertebrate definitive hosts. Those were the three representatives of the Helicidae family (and the Helicinae subfamily) in our sample, namely *C. aspersum*, *C. nemoralis*, and *T. pisana*.

Combining our present and previous [32] results, four species of *Brachylaima* may occur in Arçais. First, *B. mesostoma* can use *C. aspersum* as first intermediate host, an association found by Gérard *et al.* [32], but not in the present study. Here, we demonstrate that a second species, the unidentified *Brachylaima* sp. recorded in *C. aspersum* at a sporocyst stage in Arçais by Gérard *et al.* [32], which we termed *Bra1* here, also uses *C. aspersum* and *C. nemoralis* as second intermediate hosts. According to the morphological details, a possible third species of *Brachylaima* (*Bra1-Tp*), very similar to but smaller than *Bra1*, may occur in *T. pisana*. Finally, we recorded at a metacercarial stage a fourth *Brachylaima* species (which we termed *Bra2*), with low (*≤* 6%) prevalences in *C. nemoralis* and *C. aspersum*. Our assessment that these metacercariae belong to yet another species was based on their distinctive orange colour and relatively small size (mean total length of 815 ± 207 μm, range: 520–1,050 μm).

*Cornu aspersum* appears to be the only natural first intermediate host in Arçais for *B. mesostoma* and the unidentified *Brachylaima* sp. (*Bra1*) [32], and the main second intermediate host of *Brachylaima* spp. Based on available parasitological data, six *Brachylaima* species are known to use *C. aspersum* at least as a second intermediate host, *i.e.* five in Europe (Table 1) and one species, *B. cribbi*, in Australia [9]. The notably large size of *Bra1* metacercariae in Arçais (2,127 ± 125 μm, range: 1,739–2,430 μm) suggests that this unidentified *Brachylaima* species may be different from the other species known to use *C. aspersum* at least as a second intermediate host, including *B. aspersae* (644 μm, range: 495–882 μm), *B. llobregatensis* (1,243 μm, range: 982–1,485 μm), *B. mascomai* (1,321 μm, range: 604–,1814 μm), and *B. cribbi* (1,152 μm, range: 625–1,912 μm) ([32] for review). Moreover, the comparison of our DNA sequences with those available in GenBank also suggests that the *Bra1* species is different from *Brachylaima fuscata* (Rudolphi, 1819) Joyeux, Baer & Timon-David, 1932 and *B. mesostoma* [2, 32]. In contrast to *Bra1*, the smaller size of *Bra2* metacercariae in Arçais is not discriminant when compared to the other *Brachylaima* spp. recorded in *C. aspersum* ([32] for review). However, as far as we know, the orange colour of *Bra2* metacercariae has never been mentioned for any described *Brachylaima* species, and may suggest that it is a new species. Unfortunately, DNA sequences of *Bra2* orange metacercariae only allowed us to conclude that this species belongs to the genus *Brachylaima*; investigations are needed to clarify its exact nature.

Apart from *C. aspersum*, the only other second intermediate hosts of *Brachylaima* spp. were *C. nemoralis* and, to a lesser extent, *T. pisana*. It is notable that these two species are the most closely related to *C. aspersum* among our sample (Helicinae subfamily). This suggests that the snail features that allow the metacercarial development of closely-related *Brachylaima* species may be phylogenetically conserved.

*Cepaea nemoralis* is parasitised at similar prevalences and intensities by metacercariae from both unidentified *Bra1* and *Bra2* species (Table 3), with two snails harbouring mixed infections in their kidney. Infections with *Bra1* metacercariae were previously documented at this site [22]. In other European regions, *C. nemoralis* was also found to be infected by *Brachylaima* metacercariae, including *B. mesostoma* [2, 23, 25, 73]. So far, *B. mesostoma* is the only *Brachylaima* species positively identified in *C. nemoralis* (although several studies identified infections only to the genus level, e.g. [23]). Furthermore, *B. mesostoma* as sporocysts has previously been demonstrated in *C. aspersum* in Arçais [32]. Therefore, it is possible that some of the non-sequenced metacercariae in *C. nemoralis* here also belong to this species.

*Theba pisana*, in contrast with *C. aspersum* and *C. nemoralis*, was only found infected once by brachylaimids in our sample, with a single *Brachylaima* metacercaria in one adult. This contrasts with previous studies showing that it can be infected, at relatively high prevalence (from 10% to 75% [12, 36]), by both sporocysts and metacercariae from a range of Brachylaimidae species. This includes, in Europe, *Postharmostomum commutatum* (Dietz, 1858) (syn. *Brachylaima commutatus* according to Valadão *et al.* [70]), a poultry fluke for which *T. pisana* is the single known species as first and second intermediate host, *B. fuscata*, and *B. mascomai* [27, 36]. It is also susceptible to experimental infections by *B. llobregatensis* [40]. Moreover, *T. pisana* is also described as both first and second intermediate host for *B. cribbi* in Australia where the snail is considered invasive [13]. The metacercaria found in *T. pisana* was smaller than the other metacercariae found in Arçais (excluding the orange *Bra2*). Potential candidate species based on these measurements include *B. llobregatensis*, *B. mascomai*, *B. mesostoma*, or even the pathogen species *B. cribbi* (see [32] for review). Further studies including DNA sequencing are needed to first confirm that the *T. pisana* infection was not accidental, and then to identify which *Brachylaima* metacercariae are associated with *T. pisana* in France.

### Shell-encapsulation by land snails as a general defence against natural enemies

The encapsulation of parasites within the shell is a defence mechanism of land snails that was initially believed to be specific to nematodes [20, 28, 61], but which we previously showed can also be used against trematodes in *C. aspersum* and *C. nemoralis* (prevalence of 6.9% and 1.4%, respectively) [33]. The new findings recorded in the present study (*i.e.*, shell-trapped metacercariae and nematodes in *C. neglecta* and *P. elegans*, respectively) confirm that land snails are able to kill not only nematodes, but also trematodes by shell encapsulation, and that this ability may be phylogenetically widespread among terrestrial gastropods. Moreover, in addition to shell-trapped parasites in land snails, our discovery of dipteran larvae encased in the shell of *T. pisana* may suggest that shell-encapsulation is a more generalist defence response than was previously thought against various natural enemies including parasites, parasitoids, and invertebrate predators.

## Conclusion

The present study highlights the limited knowledge we have about host-parasite interactions involving terrestrial gastropods, a reflection of the more general knowledge gaps regarding parasite diversity [15, 17]. Importantly, even in western Europe where the malacofauna is very well described, and regarding a parasite family (Brachylaimidae) with potential impacts on vertebrate wildlife, domestic animals and even human health, our observations in a single rural garden may have unearthed undescribed parasite species, and at the very least multiple newly recorded host-parasite interactions.

Many *Brachylaima* species are only described from their vertebrate hosts [34, 41]; studies tracking trematodes in their environment across their entire life cycle (including faeces samples and how snails interact with them) are needed. Taken together, these would increase our knowledge of ecologically important but neglected interactions and how they may respond to anthropogenic environmental changes [16], provide new models for epidemiological studies in the wild, and help us better understand the risks associated with wild snail consumption.
